# Triggers of self-conscious emotions in the sexually transmitted infection testing process

**DOI:** 10.1186/1756-0500-3-229

**Published:** 2010-08-17

**Authors:** Myles Balfe, Ruairi Brugha, Diarmuid O' Donovan, Emer O' Connell, Deirdre Vaughan

**Affiliations:** 1 Department of Epidemiology and Public Health, Royal College of Surgeons in Ireland, Dublin, Ireland; 2 Department of Health Promotion, National University of Ireland Galway, Galway, Ireland

## Abstract

**Background:**

Self-conscious emotions (shame, guilt and embarrassment) are part of many individuals' experiences of seeking STI testing. These emotions can have negative impacts on individuals' interpretations of the STI testing process, their willingness to seek treatment and their willingness to inform sexual partners in light of positive STI diagnoses. Because of these impacts, researchers have called for more work to be completed on the connections between shame, guilt, embarrassment and STI testing. We examine the specific events in the STI testing process that trigger self-conscious emotions in young adults who seek STI testing; and to understand what it is about these events that triggers these emotions.

Semi-structured interviews with 30 adults (21 women, 9 men) in the Republic of Ireland.

**Findings:**

Seven specific triggers of self-conscious emotions were identified. These were: having unprotected sex, associated with the initial reason for seeking STI testing; talking to partners and peers about the intention to seek STI testing; the experience of accessing STI testing facilities and sitting in clinic waiting rooms; negative interactions with healthcare professionals; receiving a positive diagnosis of an STI; having to notify sexual partners in light of a positive STI diagnosis; and accessing healthcare settings for treatment for an STI. Self-conscious emotions were triggered in each case by a perceived threat to respondents' social identities.

**Conclusion:**

There are multiple triggers of self-conscious emotions in the STI testing process, ranging from the initial decision to seek testing, right through to the experience of accessing treatment. The role of self-conscious emotions needs to be considered in each component of service design from health promotion approaches, through facility layout to the training of all professionals involved in the STI testing process.

## Background

Sexually transmitted infections (STIs) are public health priorities because of their associated risks, such as infertility, ectopic pregnancy and pelvic inflammatory disease [1-3]. Early detection and treatment of STIs is important so as to shorten STI infection duration, which in turn determines the risk of transmission of STIs to partners and the severity of sequelae [4].

Emotions such as embarrassment, shame and guilt play important roles in influencing individuals' decisions to seek treatment for suspected STIs, and also their experiences of attending sexual health services if they choose to act on these decisions [5]. These emotions can act as significant barriers to accessing appropriate healthcare advice and treatment [5,6]. Research has shown that individuals can feel embarrassed looking at information about STI testing, which can put them off reading and following through on this information [7,8].

Embarrassment is commonly experienced by young people who talk to healthcare providers about their sexual behavior, and can result in them failing to request STI testing [9,10]. Young people can even feel ashamed just anticipating talking to a health professional about these matters [11]. Individuals often feel ashamed about being witnessed (or thinking that they might be witnessed) visiting STI clinics, which can discourage them from attending these services [12]. Being offered opportunistic screening by professionals in public areas (such as at reception desks) can be 'excuciatingly embarrassing' for some young people [13].

Those who do decide to seek STI testing often struggle to manage these feelings and may drop out of the STI testing process if they cannot do so [14]. A positive diagnosis of an STI can trigger intense negative affect [15]. These emotions can prevent young people from discussing their STI status with their past, present or future partners. This in turn increases these partners' risks of developing STI-related sequelae if they, too, are infected [9,16]. Individuals who choose to hide their positive STI status from their partners, however, often feel guilty about doing so [15].

Overall, the studies mentioned here have demonstrated that emotions such as embarrassment, shame and guilt are part of many people's experiences of seeking STI care. These feelings are deeply unpleasant to experience and individuals who seek STI testing are motivated to avoid them [8,10], even though this can have negative impacts upon their health. Consequently, researchers [8,17] have called for more work to be undertaken on the connections between shame, guilt, embarrassment and sexual behaviors, including STI testing.

In this article we explore and identify the specific events in the STI testing process that trigger emotions such as shame, embarrassment and guilt in young adults who seek STI testing; and seek to understand what it is about these events that triggers these emotions. Being able to identify specific emotion inducing-incidents and components of the STI management process should enable service managers and practitioners to design sexual health services that decrease the likelihood that patients will experience distressing emotions during their testing experience and that will consequently increase service uptake, especially among young adults who are at most risk.

## Self-conscious emotions

The emotions of embarrassment, shame and guilt share substantial features in common [8], such that researchers have argued that they form a single family of negative emotions- the self-conscious emotions. These emotions are labeled self-conscious because individuals' understandings and evaluations of their own selves, and their awareness of the selves of others, are crucial to their formation [17]. Self-conscious emotions develop when individuals' skills, competencies or characteristics are called into question or transgress relevant social standards [18], and when individuals believe that others will negatively view their failures or violations. This latter point is crucial - individuals experience self-conscious emotions not as a result of how they view themselves, but from how they think others view them; from imagining how they are viewed through the eyes of other people [18]. 'Others' can be real people, or they can be internalized standards or behavioral reference points (e.g. images of how a person is 'supposed' to behave in a particular situation) against which the person compares her own practices.

Though most self-conscious emotions are unpleasant to experience, they have important social functions [8]. For example, they help to keep behavior within acceptable social limits [19]. The negative flavors of these emotions mean that once individuals are given a taste of them, they are usually motivated to avoid situations that give rise to these emotions in the future [17]. Self-conscious emotions can also act as behavioral flags, indicating to a person instances where she is not living up to expected standards; they thereby provide the person with important feedback on her thoughts and practices as these could be viewed by others [8].

There are different types of self-conscious emotions. Shame, or self-disgust [19], is a negative emotion characterized by intense discomfort, feelings of exposure, inadequacy and worthlessness, and a desire to hide. It develops when an individual fails in relation to some personal or social standard, feels this failure to be a representative symptom of a globally defective self or identity [5] and believes that others will appraise this failure negatively [19]. Common triggers of shame include poor-performance and role-inappropriate behavior [19]. Shame is associated with a desire to undo or destroy those aspects of the self that give rise to it. Tantam [19] notes that many people will go out of their way to avoid situations where they could be shamed, because these feelings are almost like dying or being annihilated.

Guilt is an emotion that is similar to shame, though it arises in response to a specific undesirable behavior rather than in response to an undesirable self [20]. Unlike shame, situations that produce guilt do not tend to call individuals' entire selves into question. And whereas shame induces a desire to run and hide from others, guilt encourages the individual to move towards others in order to make reparations for the behavior that caused the guilty feeling in the first place.

Embarrassment, according to Miller [21], is a self-conscious emotion characterized by flustered abashment and chagrin stemming from events that increase the threat of unwanted evaluations from real or imagined audiences. As with guilt and shame, the crux of embarrassment is negative evaluation by real or imagined others [8]. Embarrassment develops when individuals believe that others have formed an undesirable impression of them because they have violated norms of demeanor, good manners, self-control or bearing [21,22]. As with guilt, though unlike shame, embarrassment is likely to develop in relation to a characteristic that an individual does not believe to be representative of her core identity. In general, individuals are less likely to become embarrassed around people they know well, because they are more certain that these people will not negatively assess them.

Self-conscious emotions are often triggered by self-presentational failings [8]. Self-presentation refers to the tactics and strategies that individuals use to control how others perceive and evaluate them [8]. By engaging in self-presentational activities individuals can construct and maintain positive social identities and can claim 'face' [8], or social worth and respect. Individuals will usually attempt to present images of themselves that are consistent with the norms that are relevant to particular social settings and with the general social roles that they occupy [18]. When individuals' execute their self-presentational activities in a flawed manner, however, for example by doing something that undermines an image of competence, or when they engage in role inappropriate behavior that is witnessed by others, self-conscious emotions can be triggered. Individuals are generally motivated to avoid potential situations that could lead to self-presentational failures and consequently generate feelings such as shame, guilt or embarrassment.

## Methods

Thirty semi-structured interviews were conducted with young adults who had previously attended health services for STI testing in the Republic of Ireland. Respondents were recruited from three General Practice (GP) practices (one urban middle-class GP practice, one urban working class GP practice and one rural GP practice), one Third Level College health service, two Family Planning clinics (one serving an urban middle-class population and the other serving an urban working class clientele) and a specialist STI treatment service for men who have sex with men (MSM). We utilized a qualitative approach because we wanted to explore young people's perspectives in detail. Qualitative methods have proven efficacy in sexual health research [23].

Respondents were eligible to take part in the study if they were between eighteen and twenty nine years of age (the age group with the greatest increase in detected incidence of STIs in the Republic of Ireland over the previous decade) [24]. We purposely sampled respondents in order to recruit both men and women, respondents of different ages (late teens, early 20 s, mid 20 s, late 20s), and respondents who had sought STI testing from specialist and community-healthcare settings. Twenty one respondents were female, nine were male. Three respondents (recruited from a specialist urban health clinic for gay men) self-identified themselves as being gay. The other twenty-seven respondents self-identified themselves as being heterosexual. We did not ask respondents to self-identify their class backgrounds, though seven respondents were recruited from urban working class settings, ten from urban middle-class settings, five from the college health service and five from the rural GP setting (which also served a primarily middle-class population). Approximately 33% of potential respondents whom we approached agreed to take part in the study (we handed out 110 recruitment leaflets to potential respondents, of whom 30 agreed to be interviewed). Ethical approval for the study was received from the authors' institutional ethics committee and from the ethics committee of the Irish College of General Practitioners.

Respondents were recruited either by the first author or by a healthcare professional working in the healthcare setting that respondents were attending for STI testing. Potential respondents were provided with an information leaflet, which provided background information on the study, what participation in the study would involve (up to one hour interview focusing on respondents' experiences of attending services for STI testing); respondents' freedom to avoid issues and stop the interview at any point; and assurances on anonymity and how information would be recorded and used. Participating respondents were asked to respond 'yes' by text if they were interested in taking part in the study and were given the option of completing their interview by telephone or face-to-face. Twenty four respondents chose to do their interview by phone and six face-to-face. Respondents who completed face-to-face interviews were asked to provide written consent to take part in the study. Telephone interviewees were asked for verbal consent to take part. Telephone interviewees were asked over the phone if they wished to take part in the study, and were reminded about guarantees around anonymity and data storage. They were informed about the overall purpose of the study and were asked to provide verbal consent if they wished to take part in it. Respondents were informed that they could stop their telephone interview at any point, and that they did not have to answer any questions that they did not wish to. They were also asked if they had any questions about the project. Once the interview began respondents were put on loudspeaker on the first author's office telephone and the interview was recorded with a digital voice-recorder (approval to record the interview was sought before the interview began). When the interview had finished the voice-recorder was turned off and respondents were asked for their home address. The interviewer posted respondents gift vouchers to this address and then deleted the address.

The first author, a Ph.D. sociologist specializing in qualitative methods, interviewed respondents. A semi-structured interview approach was used for both face-to-face and telephone interviews. Interview questions examined respondents' experiences of seeking STI-testing and specific events in the STI-testing process that respondents especially liked or disliked (and why). Follow-up questions explored the reasoning behind respondents' responses in more detail. A non-directive approach was used in the interviews to allow respondents to shape their own accounts. Interviewing continued until we deemed data saturation to have been reached (the point at which no new themes were emerging). Researchers have proposed 30 as an approximate or working number of interviews at which one could expect to be reaching theoretical saturation when using a semi-structured interview approach [25].

Interviews were tape-recorded (with respondents' permission) and fully transcribed by a medical secretary. To check the accuracy of the transcripts both authors re-listened to the interview recordings of the first three interviews while reading the transcripts of those interviews; subsequent transcripts were read without reference to the original audio recordings. Both authors were involved in coding and analyzing all of the transcripts. The analysis process was as follows: significant key words, phrases and themes in the interviews were marked with summary words or codes. As each transcript was coded, all codes that were thematically similar were grouped together, and labeled with a summary code, called a category. In line with standard qualitative procedures, these categories then became the organizing themes of our analysis. Categories related to instances in the STI testing process that triggered self-conscious emotions for respondents. Categories were not pre-determined but emerged during analysis. For example, when we began the study we had a fairly open-ended objective: to examine the barriers that prevented or discouraged young adults in Ireland from seeking STI-testing. It was only when we began conducting the interviews, and particularly when we began analyzing them, that we saw the central role played by self-conscious emotions (particularly shame and embarrassment) in respondents' experiences. In situations where discrepancies were detected between codes the procedure was for both authors to meet to discuss these discrepancies; and to discuss whether or not the discrepancy existed because codes addressed different aspects of respondents' experiences (i.e. different events in the STI testing process) or because we had not yet constructed a sufficiently abstract category under which apparently different codes could be sublimated (i.e. the codes appeared to be superficially different, but were actually different 'symptoms' of the same underlying process).

In the findings section representative quotations are accompanied by codes providing details about the respondents who uttered those quotations (see Table 1 for a key to these codes).

**Table 1:** Key for respondent quotation codes

Key for respondent quotation codes	
M	Male

F	Female

Early/mid/late 20s	Respondents' age

SH	Went for STI testing in Student health service

FP	Went for STI testing in Family Planning service

GP	Went for STI testing in GP practice

STI	Went for STI testing in STI clinic

MSM	Went for STI testing in specialist clinic for men who have sex with men (MSM)

-	Tested negative for STI

+	Tested positive for STI

## Results

### Reason for seeking STI testing as a trigger for self-conscious emotion

Respondents sought STI testing for one of four reasons: 1). They had unusual symptoms near their genital regions; 2). They were required to by an employer; 3). They were in a sexual relationship and being about to stop using condoms; and 4). They had had unprotected sex with a stranger. Respondents who sought STI testing for the first, third and fourth of these reasons experienced anxieties about their risk status (i.e. about whether or not they had an STI), which prompted them to seek testing. These respondents did not, however, associate significant shame or embarrassment with their decision to seek testing.

I was up the walls. Completely up the walls. Just because I' m a worrier by nature. I'd just sit there and be like, 'oh God, the organisms are multiplying, it's getting worse'. (F/late 20s/urban GP/-).

Chlamydia is such a scary, scary thing now, because it's, you know, asymptomatic (F/late 20s/urban GP/-).

I have always been scared shitless of getting something like HIV or even syphilis or genital warts and I remember in school they'd show us pictures of black rot and stuff like that and that would just scare the shit out of you. So I had to go. (M/early 20s/student health GP/-).

Respondents who sought STI testing because they had had unprotected sex also felt anxious about their risk status.

That time because I knew I'd had unprotected sex I was quite unnerved by the fact that I could have something. (F/early 20s/urban GP/-).

However they also felt ashamed and embarrassed at the behaviors that had led them to seek STI testing, principally because they felt that these behaviors violated salient social norms governing how sexual risk was supposed to be managed (i.e. responsibly and carefully).

It's the whole embarrassment, the shame because you made a mistake you know. (F/late 20s/urban GP/-).

I felt ashamed first because you know you should be taking protection. You know it's [unprotected sex] something you' re not supposed to do you know. (F/early 20s/urban GP/-).

### Embarrassment associated with talking to peers and partners about STI testing

Once they had decided to seek STI testing, respondents (whatever their initial reason for deciding to seek STI testing) were faced with another decision: whether or not to inform their partners and peers about their intention to go for testing. Many respondents felt embarrassed about the thought of doing so, concerned that their social identities would become discredited and their partners or peers would view them negatively.

Interviewer: Do you think you would have ever been the one to bring testing up with your partner first?

Respondent: No. I'd have felt shy and a bit embarrassed (M/early 20s/student health GP/-).

I suppose it's embarrassing to bring it up. You feel kind of dirty or something. (F/mid 20s/rural GP/-).

Respondents found it easier to talk to partners and friends whom they trusted and were close to. They felt that these people would not negatively evaluate them, and so respondents' concerns about embarrassment or identity threat did not become significant barriers to communication. Respondents were most likely to keep information from friends whom they knew but did not know well, and whom they could not be sure would not judge them.

It is notable, however, that even respondents who talked to their friends about their decision to seek STI testing could find it embarrassing to do so, particularly at first. Some respondents employed distancing strategies when talking to their friends about STI testing. One such strategy was to describe STI testing as an action that respondents had engaged in in the past. Consequently, testing was less likely to be seen by respondents as something on which their current social identities could be negatively assessed.

You'd think that girls would talk a lot about things like that [STIs]. But as a group we' re actually quite embarrassed about that kind of thing and we would mainly talk past tense about that kind of stuff. We say 'oh yeah, that happened to me too'. I'd never consider telling them it's happening right now. (F/late 20s/urban GP/-).

Keeping this information secret from partners was more difficult, though some respondents did so. Even respondents who talked to their partners about their need to go for testing, however, could feel embarrassed about having these conversations.

He [partner] just said he wanted to go [for testing]. I didn' t ask him about his experience. I was more kind of embarrassed about my own experience. (F/late 20s/Family planning/-).

Most respondents reported having sexual partners of approximately the same age as themselves. One female respondent, however, reported having a sexual partner who was significantly older than herself. She also reported that the age difference between herself and her partner inhibited her willingness, and increased her embarrassment, about talking to her partner about STI testing.

The respondent indicated that she had learned to play a role as a relatively powerless, desexualized innocent in her relationship with her partner, whom she represented as an older, judgmental, male authority figure. She felt that communicating to this partner that she was in need of STI testing would be an assault on the virtuous, feminine identity that she had previously established with him. She was reluctant to let this happen, which accounted for the feelings of discomfort that she experienced in relation to talking to her partner about testing.

The guy I' m with is older than me, he's a business man. I' m a student. I'd feel more uncomfortable talking to him about it. You'd be making suggestions about yourself too. You' re trying to come across as a good girl and then maybe all [sexual activities engaged in the past] hasn' t been so good in the past. (F/late 20s/family planning/+).

### Shame and embarrassment associated with accessing STI testing facilities

Accessing healthcare settings for testing was considered to be an embarrassing event by many repondents. Walking into a setting and making an appointment with reception staff was an instance where respondents might have to reveal discrediting information about themselves to strangers such as reception staff and passers-by. Respondents often felt self-conscious about revealing this information to these people.

Respondents who were required to state the reason for their appointment to reception staff, and respondents who went for testing in healthcare settings whose sole purpose was to offer STI testing, reported experiencing greater embarrassment than respondents who did not have to reveal the reason for their appointment to staff receptionists. Settings that offered multiple health services apart from STI testing (such as GO practices) afforded respondents more anonymity. Respondents could be attending these settings for any reason, and so these settings were less likely to trigger self-conscious emotions in respondents.

I just went away and made the appointment in the clinic and I kind of felt a bit awkward because I was slightly embarrassed. You kind of felt a bit like you shouldn' t be sitting here in the first place. (F/late teens/student health/-).

There's just such a stigma attached to it that you know, I can' t go in case somebody sees me. There's an awful fear of 'what will people think'. (F/mid 20s/STI/+).

Several respondents reported that anticipated embarrassment influenced their decision about where to seek STI testing. These respondents thought (prior to seeking testing) that certain settings would be more likely to embarrass them than others (because these settings lacked identity-protecting orientations); these 'embarrassment-prone' settings were subsequently avoided in favor of settings where respondents thought that they would feel more comfortable and less self-conscious.

Well I did consider going to the specialist clinic but I never wanted to go. I'd put it off. Because of the fear of how you'd feel being over there. (M/late 20s/urban GP/-).

Interviewer: So if you had gone to the specialist clinic would you have felt self- conscious?

Respondent: Definitely, yes. Which like I was saying is one of the reasons why I didn' t go (F/mid 20s/urban GP/-).

Sitting in clinic waiting rooms was considered to be an unpleasant activity by many respondents, particularly those attending specialist STI settings. Respondents felt that merely being in these rooms undermined their identities, transmitted discrediting information about them to other people in the waiting areas, for example other individuals waiting for testing, receptionists, porters and even individuals walking by the clinic. This realization could trigger strong feelings of discomfort and self-consciousness in respondents.

The waiting room seemed really seemed dark and foreboding, I suppose, and you feel like shameful as well. (F/late 20s/STI/-).

It kind of feels, I don' t know, it feels dirty a little bit. They' re all looking at you as if to say well 'I know what he's here for' (M/late 20s/urban GP/-).

The social characteristics of who were waiting in the clinic reception areas played a role in determining the degree of self-consciousness that respondents experienced while they waited to be called for testing. Self-consciousness tended to increase if respondents felt that clinic audiences were people 'unlike themselves' (opposite age/gender/sexuality), and were consequently more likely to judge or assess them.

I had to sit in the waiting room, everyone knows I was there for an STI. It was just a bit embarrassing. There was a lot of men there, a lot of older men and that was embarrassing. (F/early 20s/STI/+).

There was four young fellas sitting there. I felt a bit embarrassed cos I was the only girl there. (F/early 20s/Ballymun/+).

Conversely, respondents' feelings of embarrassment were likely to attenuate if they felt that people in the waiting room were 'people like themselves' (same gender/age/sexual orientation), and likely to have supportive orientations towards respondents.

Like everyone's the same there. It's a gay man's project. You' re all there for the same reason. You' re only going up there to get tested. There's nothing embarrassing about it (M/late 20s/MSM/-).

You just feel more comfortable waiting when it's only women. You' re not going to run into somebody you know, especially a male. (F/late 20s/STI/+).

There was evidence that clinic staff who made efforts to make respondents feel comfortable, and indicate to respondents that they would not be negatively evaluated, could reduce respondents' feelings of embarrassment and shame.

I guess there's a certain openness in that clinic, there's nothing cloak and dagger about it. The guy who seems to run the operation down there seems to be on the ball and makes people feel comfortable. And also, on a personal note, I don' t feel embarrassed being somewhere like that. The staff are incredibly friendly. (M/late 20s/MSM/+).

Respondents who attended multi-purpose settings such as general practices generally felt less self-conscious while waiting to be called for their tests than did their counterparts who attended specialist clinics. In the former, 'audience members', i.e. other patients and receptionists, did not know the reasons why respondents were attending these settings. Respondents' identities were therefore not under threat to the same degree as they would have been in more openly 'discredited' [22] settings and self-conscious emotions were less likely to emerge.

On occasions, however, respondents attending these settings could be forced to account for their presence within them, for example because they encountered someone whom they knew in the waiting room. Such forced accountings were often described as embarrassing incidents, because they threatened to undermine respondents' identities in front of other people in the waiting areas.

I was collecting my results. And I saw this guy who I knew. I was like 'oh God, of all things, this is just great'. And he just kind of roared it out. A room full of people. 'What are you doing here? You should be at the doctor on the other campus'. I felt like dying (F/late teens/student health/-).

To help manage feelings of embarrassment and shame, many respondents (particularly female respondents) brought close friends or partners with them when they went for testing. Peers and partners helped respondents to define their STI testing activities as normal, healthy (and even fun) activities to engage in, rather than as deviant practices. Partners and peers could provide identity support to respondents, highlighting that others thought positively about them and that respondents were as 'normal' as everyone else.

I kind of went [for testing] for a giggle with some of the girls. Because it's free and it's so anonymous it's something that you can just do. (F/early 20s/urban GP/-).

I think because I' ve gone with a couple of friends, that feeling of shame isn' t there. (F/early 20s/student health GP/-).

### Shame and embarrassment experienced by respondents during interactions with healthcare professionals

Interactions with healthcare professionals had the potential to induce feelings of shame and embarrassment in respondents. They were often worried that healthcare professionals would negatively evaluate them (for being promiscuous, for being careless and for needing to engage in a stigmatized activity such as STI testing), and thereby shame or embarrass them.

The worst thing about going for testing is facing your fears. Facing a fear of having to first of all, be judged. You have this feeling it's going to be cringy [embarrassing/shameful] and this person might pass a comment on you. (F/late 20s/family planning/+).

Nevertheless, about two thirds of respondents reported having positive interactions with healthcare professionals (particularly with professionals who were of the same age/gender as themselves), which served to reduce these respondents' feelings of self-consciousness. Positive interactions were characterized by non-judgmental and accepting stances on the part of treating healthcare professionals.

About one third of respondents, however, reported having negative interactions with healthcare professionals (irrespective of whether these professionals were of the same gender or age as respondents) that served to heighten their feelings of self-consciousness. The reasons for negative interactions were similar in each case: the healthcare professional was perceived to have judged respondents.

As soon as I mentioned I was single her attitude changed and she was completely horrible and basically gave out to me despite the fact I was always [sexually] careful [used condoms]. Basically treated me like a complete idiot. I was treated like dirt. Horrible, horrible experience. (F/mid 20s/FP/+).

Every question she asked me she kind of looked at me like, like 'don' t lie to me'. Someone is knit picking at your personal life. It's a bit daunting. You kind of feel guilty that you were even having sex in the first place, never mind sitting there for an STI test. (F/late teens/DIT/-).

Respondents used a variety of impression management activities to protect their identities and manage their feelings of self-consciousness during negative interactions with jusdgemental healthcare professionals,, such as downplaying the number of sexual partners that they had had (so as to present themselves as individuals who were not careless, promiscuous 'sluts' ).

If the person whose asking you 'how many partners do you have?' seem a bit serious and looking down on you, that puts you off answering properly. You' ll kind of reduce the number by half or a third, whatever. Just to make yourself look better (F/late 20s/family planning/-).

The methods that healthcare professionals used to test for STIs also determined the intensity of the feelings of shame and embarrassment that respondents' experienced. Urine tests and blood tests were considered to be the least embarrassing tests to take; respondents could take these tests in private, or only have to expose 'public' parts of their bodies such as their arms to the treating healthcare professional.

A urine test is not that bad cos you just go to the toilet and do it yourself (F/early 20s/urban GP/-).

Female respondents who took a cervical swab test, however, usually found the experience to be *embarrassing*; cervical swab tests violated norms of cleanliness, modesty and privacy that recommend that women do not reveal their 'dirty private parts' [10] to strangers.

She [doctor] kind of just says 'ok, go in there, take off your clothes and lie up on the bed'. It's embarrassing having somebody you don' t know do that. (F/early 20s/fp/-).

A minority of respondents who took a cervical swab test reported feeling *shamed *(rather than embarrassed) by the experience. Shame was triggered in these respondents not only by the tests' violation of modesty norms, but also by these respondents' beliefs that they had violated norms of sexual morality and responsibility. Interestingly, some of these respondents appeared to welcome their shame, and wanted to use this shame to hurt or damage the immoral and irresponsible sides of themselves that led them to take a cervical swab exam in the first place.

I had to go in there, take off my knickers and get up on the table. I kind of felt disgusted like I shouldn' t be having sex in the first place. I shouldn' t be getting into those situations. I shouldn' t be sexually active. Basically I just felt a no sex before marriage kind of policy coming off my head. I kind of felt disgusted that way. (F/late teens/SH/-).

Respondent: The experience was very uncomfortable, up on the table and that. It was very humiliating and embarrassing. But like, my thoughts on it would I was stupid enough to have unprotected sex, 'good enough to you, like'. So therefore I had to go through the shame of admitting to it and how silly I'd been and the lying up on the table getting swabbed and that.

Interviewer: So almost like it was kind of punishment in some way.

R: Yeah, exactly. (F/mid 20s/rural GP/-).

### Test positive or negative? How results affect identity

Respondents who tested negative for an STI, or who tested positive for an STI that they felt had no particular stigmatizing connotations, usually felt proud that they had sought STI testing.

It was NSU. Not too serious. So I felt good that I'd gotten the test and found out about it. (M/early 20s/SH/+).

Respondents who tested positive for an STI that had stigmatizing connotations, however, usually felt ashamed (though for some this feeling lasted for only a brief period of time).

I felt so ashamed of myself. I was like 'God, how could I get an STI?' I never would have thought a year ago, two years ago, that I would get an STI. How could this happen to me, how could I be so careless. (F/early 20s/STI/+).

I was mortified. I sat and I think I cried for about two hours when I got home. I felt disgusted and embarrassed and very foolish. (F/late 20s/GP/+).

There was evidence that shame and self-disgust was stronger in respondents who had been diagnosed with STIs that affected their appearance.

It's the fact that warts changes what you look like. You' ve a constant reminder of 'that's disgusting'. You feel a bit like...it's just the stigma. Something like Chlamydia is a clean disease. You don' t see anything. It's just there. Like herpes, genital lice, genital warts, it's unsightly. (F/early 20s/STI/+).

Being diagnosed with an STI could have negative impacts on respondents' sexual practices. This tended to happen when respondents' internalized negative ideas of what a person with an STI 'was', which led them to experience an 'altered shame-based self-definition' [26] and lose their previously held 'respectable' self-concept.

Respondent: After I was diagnosed I basically didn' t care any more and started having one night stands for about a year after. I went off the rails and became really slutty. It did make me think I must be one of 'those' girls if I got Chlamydia. It made me think I was a slut even though I wasn' t and I ended up acting that out because I thought, it must be true if I have an STI. I wouldn' t have put myself in that category and I was like 'I must be in that category, or I wouldn' t have got this'. (F/mid 20s/GUM/+).

### Notifying partners and others as an emotional trigger

Notifying sexual partners (past and present) about a positive STI diagnosis was considered to be an embarrassing activity by most respondents.

I'd just be absolutely mortified. It would really depend on the person. If they said to me go and tell those two people you had a one night stand with, then I'd be like absolutely no way. (F/early 20s/STI/+).

And then having to sit down and make the call to them, that's where the embarrassment came in. (F/late 20s/rural GP/+).

A minority of respondents indicated that they would not inform their partners about positive STI diagnoses so as to avoid having embarrassing confrontations with them. The majority of respondents, however, indicated that they would inform their partners about their diagnoses. These respondents described partner notification as a correct, responsible practice to engage in, and indicated that they would feel guilty if they did not engage in it.

Female respondents experienced feelings of guilt particularly strongly; these respondents' feelings were triggered by thoughts of how STIs could affect their partners' future girlfriends (i.e. guilt was directed towards individuals these respondents could easily empathize with).

I had to pick up the phone and speak to him [ex-partner] about it. I don' t think I could ever forgive myself if in five or ten years time an ex-partner's partner couldn' t have children because she'd had Chlamydia for so many years and never knew. As a woman I could never forgive myself. (F/late 20s/GP/+).

I would feel enormous guilt if I gave somebody else an STI and I didn' t tell them about it, I really would. I would feel I had put a huge burden on them that they had to deal with (F/early 20s/STI clinic/+).

Respondents also described feeling ashamed at the thought of informing their parents and friends that they had been diagnosed with an STI.

I was afraid my parents would be ashamed of me. I was already not feeling too brilliant about myself, so I was worried that my parents would be disappointed in me and ashamed of me. (F/early 20s/GP/-).

Some respondents avoided informing other individuals about their diagnosis because they felt that these individuals would attempt to take ownership of their experiences, and prevent respondents from processing their feelings of shame and embarrassment.

I didn' t want to tell my mother. I just knew the way she'd react. Whenever anything bad happens she's too pragmatic about it. Just get up and brush it off. Sometimes you can' t deal with that. You' re like, no, 'let me be upset. I am ashamed'. (F/early 20s/STI/+).

### STI Treatment

Respondents who tested positive for an STI, and whose treatment regimen consisted of taking a single dose of medication (for example, for Chlamydia) from the treating healthcare professional reported less treatment-orientated shame and embarrassment than respondents who had to return multiple times to their healthcare clinic for treatment (for example, because they had a viral STI such as Herpes), or who had to access treatment from public pharmacies.

I had to go in [to the hospital every month [for treatment for Herpes] and it was painful and embarrassing. (F/late 20s/STI/+).

Respondents who obtained treatment from pharmacies experienced embarrassment for much the same reasons that respondents accessing healthcare settings for testing did: they might have had to reveal discrediting information about themselves to people whom they did not know or trust. These respondents therefore engaged in a variety of impression management activities in order to minimize the risks of other people finding out about their diagnoses, such as seeking treatment from pharmacies that they would not usually attend.

My friend works in a chemist in town and I didn' t go to that one. I did go to another one in town. I didn' t want to go into her chemist cos I know the people there. I wasn' t comfortable there. (M/late 20s/MSM/+).

Respondents whose treatment lasted over an extended period of time, for example because they had been diagnosed with viral STIs, needed to manage ongoing feelings of shame and self-consciousness. In order to emotionally cope with their experiences, these respondents described needing to distance and dissociate themselves from their treatment activities.

It was very clinical going for the medication. I'd go to the hospital at this time every whatever. Get the bus, go in, make sure it doesn' t clash with any lectures. Get there early, be one of the first people there all that kind of thing. Then forget about it for another month. Just make sure I used the cream they gave me. If I was thinking about it a lot I would have gone mad. I just had to become automatic. I knew I had to get rid of it but again it was a bit like no one else is going to do it for you. I don' t know (starts crying). I don' t know why I' m getting upset, it was a year ago. But it's never just a normal trip to the clinic for anyone. There's always going to be deep emotions involved for people. (F/early 20s/STI/+).

## Discussion

This is one of only a small number of empirical studies [5,8,9,27] to investigate the events that trigger self-conscious emotions in young adults who seek STI testing, although these is an increasing realization that emotional factors play important roles in young people's STI testing experiences [5]. Several of our findings are, as far as we are aware, original in terms of previous international research on this topic, including; that young people may feel more embarrassed about having to inform older partners than younger ones about the fact that they have sought STI testing; that going for STI testing with peers can reduce feelings of embarrassment for young women; that STIs that affect appearances can induce greater feelings of self-consciousness in young people than STIs that do not; that the shame associated with testing positive for an STI can negatively impact upon young people's personal identities and lead to them to engage in risky sexual practices; that some young people are concerned about informing other individuals about their STI testing practices because they believe that these individuals will not let young people process their feelings of shame and embarrassment; and that self-conscious emotions can have a particularly pernicious effect on young people whose treatment for an STI extends over a priod of time.

The relatively small sample size (30) and the fact that most of the interviews (24) were conducted over the telephone could be considered limitations, the latter because of the inability to see non-verbal responses that could guide follow-up questioning. However, by the time 30 interviews had been completed, all of the themes that had emerged were being reproduced in later interviews. Also, our experience was that the young respondents were quite comfortable with telephone interviews and that interviewing by telephone allowed respondents greater anonymity, which facilitated additional information emerging than might have been the case if the interviews had been face-to-face.

We identified seven specific triggers of self-conscious emotions in respondents' accounts and consider here their implications for the design of sexual health services for young people. The first of these relates to *respondents' reasons for seeking STI testing*. Having unprotected sex was the most likely factor to generate embarrassment and shame in respondents. This appeared to be because respondents who had had unprotected sex felt that their behavior violated social norms governing how sexual risk was supposed to be managed (i.e. carefully and responsibly), whereas respondents who had sought testing for other reasons did not believe that their behaviors violated these norms. Previous studies have also noted that young people can become embarrassed or feel guilty if they feel that they have engaged in (what they believe to be) careless or irresponsible sexual activities [16].

The second trigger of self-conscious emotion in respondents' accounts was *having to inform peers and partners about the need to seek STI testing*. Previous work has noted that young people often feel embarrassed about talking to their peers and partners about STI testing [9,16]. The degree of embarrassment that respondents in this study anticipated experiencing was related to the perceived likelihood that they would be judged or negatively assessed by their partners or peers. It is potentially worrying that some respondents would consider not informing their partners that they had sought STI testing, for while this would protect respondents from experiencing shame or embarrassment it would increase the risk of partners suffering from STI-related sequelae if these partners had already been infected with undiagnosed STIs. We have not seen the finding been previously reported that young people might feel embarrassed about informing older partners. It is interesting, however, as it suggests that young adults who are involved in sexual relationships characterized by perceived unequal relations of power may inadvertently place their more 'powerful' partners at risk in an effort to avoid self-consciousness and threats to their identities. This also perhaps indicates a need for young people to be taught (e.g. in school) how to negotiate sexual health matters related to STI testing with partners, irrespective of these partner's ages.

The third trigger was *accessing STI testing facilities*. Many respondents indicated that they felt embarrassed about entering clinics and sitting in waiting areas. This was especially the case for those attending specialist STI settings. Previous research supports this finding [6,13,16]. What has been less commonly reported in the international literature, however, and what we found in this study, is that concerns about anticipated embarrassment influenced young adults' decisions about where to seek STI testing. Respondents' anticipated concerns were usually based on pre-conceptions rather than on previous direct experience of using clinics. We also found that bringing peers along for testing could reduce young adults' experiences of felt embarrassment while they were attending STI testing facilities (by enabling them to define the testing activity as a 'normal' rather than 'deviant' experience); we have not seen this finding previously reported.

The findings related to the third trigger firstly point to the value of delivering sexual health services, including STI testing and screening, in one-stop-shop general practice settings. Fear of stigma associated with attending a designated STI specialist clinic (be it well-founded or not) was a deterrent to seeking care for their sexual health needs for a high proportion of young people in this sample. This finding makes the case for implementing STI testing and screening in primary care settings as an additional strategy to specialist centres. Secondly, the findings point to the importance of the design of waiting areas, so as to minimize the likelihood of embarrassing interactions with others - other clients, staff and passsers-by - for those waiting to see a STI healthcare provider. Thirdly, where a range of acceptable non-stigmatizing services are made available, health promotion strategies need to communicate this to young people and could encourage them to bring a trusted peer as an active support and companion through the testing process. It is also positive to observe that efforts made by staff could help to reduce feelings of shame and embarrassment.

*Discrediting interactions with healthcare professionals *were a fourth trigger of self-conscious emotions. Respondents indicated that they could become ashamed and embarrassed if they felt that healthcare professionals were disparaging their identities. Negative interaction between patients and healthcare professionals is a commonly reported elicitor of self-conscious emotions in the international literature [13,11]. Royer and Zahner [9] note that young adults can also sometimes feel embarrassed to admit to their doctor or nurse that they lack an understanding of STIs, though this finding was not reported by respondents in our study. The respondents' reports of very positive as well as quite negative experiences associated with STI providers, which were usually not determined by shared sex and age, points to the need for provider training. Where providers are unable to learn and communicate acceptance and empathy to young people effectively, they should not be considered suitable for this work.

We also found that the method that healthcare professionals used to test for STIs could induce feelings of shame and embarrassment in respondents. Other studies have also noted that young people can have strong negative emotional responses to urethral swabs and pelvic examinations [4,13,14]. We have not seen it previously reported, however, that young adults can sometimes experience the shame and pain generated by these testing methods as a justifiable punishment of the behavior that forced them to seek STI testing in the first place. This finding does fit with theoretical research on self-conscious emotions, though, which suggests that individuals who experience feelings of shame often want to damage or destroy the parts of themselves that gave rise to these emotions [19].

The fifth elicitor of self-conscious emotions in respondents' accounts was receiving a positive diagnosis of an STI. Respondents who tested positive for an STI often described feeling ashamed and embarrassed at the result. Previous studies have reported similar findings [16,28,29]. This research indicates that young adults often have negative images in their minds of the 'types' of people who contract sexually transmitted infections [12,27]. When they contract an STI themselves, therefore, young people are forced to process the fact that their own selves may be 'defective', at least with respect to the cultural norms to which they subscribe. In contrast to previous studies, we did not find significant gender differences reported in emotions related to STIs. Royer and Zahner [9], for example, found that women often feel a greater sense of shame after being diagnosed with an STI than men do. We did find, however, greater feelings of shame and embarrassment amongst respondents who had been diagnosed with STIs that affected their appearances, such as herpes, than among those who had been diagnosed with 'invisible' STIs such as Chlamydia. This might be because visible STIs would more dramatically undermine respondents' attempts to present positive images of themselves in front of their sexual partners. One of the most interesting findings from this study was that intense shame (in this study, of the kind triggered by a positive diagnosis of an STI) can encourage (some) young people to engage in highly risky sexual practices. It is likely that this shame is driving these individuals to engage in activities that they feel will extinguish or damage 'unbearable' aspects of themselves. Though not addressed by this study, it may well be that internalized shame/homophobia encourages at least some young people risk groups such as young men in the MSM community to engage in risky/punishing sexual activities; this is an area that warrants further exploration.

The sixth trigger of shame and embarrassment in respondents' accounts was *having to notify sexual partners about a positive diagnosis of an STI*. Respondents often felt concerned about informing their partners about a positive diagnosis, worried that their partners would react negatively to this information. Previous studies, too, suggest that young adults can feel uncomfortable informing their sexual partners about a positive diagnosis of an STI [15,28]. Interestingly, it was another self-conscious emotion, that is guilt, that encouraged most respondents to inform their partners when emotions such embarrassment discouraged them from doing so. Respondents were also concerned about informing their parents about positive STI diagnoses. In large part this was to avoid embarrassment, but some respondents were also worried that their parents would attempt to take ownership of their emotional experience, not allowing them the space and time to process their own feelings. We have not seen this finding previously reported in the international literature.

The seventh, and final, trigger of self-conscious emotions in respondents' accounts was *seeking treatment for a positive STI*. Respondents indicated that they would use a variety of 'information control' practices to protect their identities and to reduce their risk of experiencing embarrassment while they sought treatment, such as seeking treatment from a pharmacy that they would not usually attend. Previous studies, too, have found that young adults seeking STI treatment often use cover stories to hide the shame of having an STI from audience members [15]. Here, as at each of the earlier stages from the initial decision to seek information on how to access professional help, anonymity is paramount for young people.

Threaded throughout all of the triggers of self-conscious emotion identified here are concerns on the part of respondents about being judged by other people for being sexually promiscuous or for engaging in the 'wrong' role-inappropriate practices (such as having sex before marriage). Similar concerns have been identified in previous studies [28]. These studies (and this one) reveal that self-conscious emotions often emerge in the STI testing process when individuals believe (or imagine) that others have formed undesirable impressions of them [8]. In this study self-conscious emotions generally emerged when respondents felt that their social identities were under threat.

As noted previously, self-conscious emotions serve a variety of functions in individuals' daily lives, for example calling attention to important events (e.g. that an identity is under attack) and directing behavior along socially acceptable lines. The flavor of these emotions is so unpalatable that, once given a taste, individuals are usually motivated to avoid them in the future [19]. Unfortunately, although these emotions can have positive effects in the STI testing process (for example, guilt can encourage young adults to inform their sexual partners in light of a positive STI diagnosis), overall they appear to be have mainly negative effects. In order to avoid self-conscious emotions and the self-presentational failings that give rise to these emotions, young adults often engage in practices that are risky to their own health or the health of their sexual partners, such as refusing to inform their partners that they are thinking of seeking STI testing [8].

Finally, one of the interesting findings to emerge from this study was the relative homogeneity that existed between respondents in terms of the items that triggered self-conscious emotions in them (though the degree of the self-consciousness that particular respondents associated with particular triggers could vary), irrespective of whether or not they were male or female. Nevertheless, respondents did differ along a variety of dimensions. Respondents differed in relation to: their rationale for seeking STI testing (respondents who did not seek STI testing for personal reasons generally did not experience self-consciousness during the testing process); the age of their partners (there was evidence that respondents felt more embarrassed and ashamed about informing older partners than they did about informing partners of their own ages); the settings in which respondents accessed for STI testing (those who sought STI testing from specialist settings experiencing more self-consciousness than those who sought testing from GP settings); the testing method that was used on them (women who had to take cervical swabs feeling more embarrassed and ashamed than women who only had to take urine tests); whether or not respondents tested positive for STIs (with those who had feeling more ashamed than those who had not); whether or not respondents tested positive for STIs that impacted upon their appearances (with those who had experiencing greater self-consciousness); partner notification (one area where male and female respondents differed was in relation to how they felt about the thought of not informing their partners, with female respondents indicating that they would experience more guilt than males if they did not do so); the length of treatment for their STIs (with respondents whose treatment went on for a significant period of time experiencing more embarrassment and shame than respondents whose STI could be treated with a significant doss). As noted earlier, the overall theme of this article 'self-conscious emotions act as significant barriers to testing' emerged during the period in which the study was being conducted; and as such we did not originally set out to relate self-conscious emotions to specific demographic variables. It may well be that future studies, building on the work presented here, will be able to do this and explore whether or not self-consciousness can be related to specific demographic variables for young adults seeking STI testing in Ireland.

## Conclusion

The findings of this study suggest that there are multiple triggers of self-conscious emotions in the STI testing process, ranging from the initial decision to seek testing, right through to the experience of accessing treatment. These emotions can have negative impacts on individuals' willingness to seek treatment, to inform their partners about positive diagnoses of STIs, and on young adults' interpretations of the STI testing process. The role of self-conscious emotions therefore needs to be given consideration in policy development and service provision [8], as outlined above and summarized here. The findings of this study suggest several areas for public health and health service interventions to reduce young adults' feelings of self-consciousness in the STI testing process. Where implemented, they should then be promoted in ways that will reach young people.

Firstly, the health services should ensure that STI testing is anonymous. It might also be useful for efforts to be made to encourage young people to attend for testing with their peers. A variety of settings, generalist and specialist, should be offered and easily accessible. Secondly, healthcare professionals should be trained and audited for the appropriateness of their interactions with young adults attending their clinics. Strategies to reduce distress should be considered, such as allowing young adults complete answers on their sexual history in their own time on a questionnaire sheet.

Thirdly, non-invasive detection methods such as urine testing for STIs such as Chlamydia should be offered where this is appropriate, for example in initial screening. This would facilitate screening in a wider range of non-clinical as well as clinical settings. Fourthly, seeing the same healthcare professional from initial meeting through to treatment, where possible, would help build trust and help to reduce the shame and embarrassment that young adults often feel during the STI testing process. Such measures could help make the STI testing experience a more positive one for young adults and increase their willingness to become ambassadors for STI testing and sexual health services in the future.

## Competing interests

The authors declare that they have no competing interests.

## Authors' contributions

MB designed the study, carried out all of the interviews, analyzed all of the interviews and drafted the manuscript (apart from the discussion and the conclusion). RB designed the study, analyzed all of the interviews and drafted the discussion and the conclusion. EO' C, DO' D and DV designed the study and provided feedback on the drafted manuscript. All of the authors read and approved the final manuscript.
